# Innovative strategies for the elimination of viral hepatitis at a national level: A country case series

**DOI:** 10.1111/liv.14222

**Published:** 2019-09-04

**Authors:** Sophia E. Schröeder, Alisa Pedrana, Nick Scott, David Wilson, Christian Kuschel, Lisa Aufegger, Rifat Atun, Ricardo Baptista‐Leite, Maia Butsashvili, Manal El‐Sayed, Aneley Getahun, Saeed Hamid, Radi Hammad, Ellen ‘t Hoen, Sharon J. Hutchinson, Jeffrey V. Lazarus, Olufunmilayo Lesi, Wangsheng Li, Rosmawati Binti Mohamed, Sigurdur Olafsson, Raquel Peck, Annette H. Sohn, Mark Sonderup, Catherine W. Spearman, Tracy Swan, Mark Thursz, Tim Walker, Margaret Hellard, Jessica Howell

**Affiliations:** ^1^ Burnet Institute Melbourne VIC Australia; ^2^ School of Public Health and Preventive Medicine Monash University Melbourne VIC Australia; ^3^ Centre for Health Policy Imperial College London London UK; ^4^ Harvard T H Chan School of Public Health Harvard University Boston MA USA; ^5^ Universidade Catolica Portuguesa Lisbon Portugal; ^6^ Faculty of Health, Medicine and Life Sciences Maastricht University Maastricht the Netherlands; ^7^ Health Research Union Tbilisi Georgia; ^8^ Department of Pediatrics and Clinical Research Center Ain Shams University Cairo Egypt; ^9^ School of Public Health and Primary Care Fiji National University Suva Fiji; ^10^ Aga Khan University Karachi Pakistan; ^11^ Ministry of Health and Population Cairo Egypt; ^12^ Global Health Unit University Medical Centre Groningen the Netherlands; ^13^ Medicines Law & Policy Amsterdam the Netherlands; ^14^ School of Health and Life Sciences Glasgow Caledonian University Glasgow UK; ^15^ Health Protection Scotland Meridian Court Glasgow UK; ^16^ Barcelona Institute for Global Health (ISGlobal) Hospital Clinic University of Barcelona Barcelona Spain; ^17^ University of Lagos Lagos Nigeria; ^18^ ZeShan Foundation Wanchai Hong Kong; ^19^ Department of Medicine University of Malaysia Kuala Lumpur Malaysia; ^20^ Gastroenterology and Hepatology Landspitali University Hospital Reykjavik Iceland; ^21^ World Hepatitis Alliance London UK; ^22^ TREAT Asia/amfAR Foundation for AIDS Research Bangkok Thailand; ^23^ Division of Hepatology Department of Medicine Faculty of Health Sciences University of Cape Town Cape Town South Africa; ^24^ Treatment Action Group New York NY USA; ^25^ Department of Hepatology Imperial College London London UK; ^26^ Department of Gastroenterology and General Medicine Calvary Mater Newcastle NSW Australia; ^27^ School of Medicine and Public Health University of Newcastle Newcastle NSW Australia; ^28^ Hepatitis Services Department of Infectious Diseases The Alfred Hospital Melbourne VIC Australia; ^29^ Doherty Institute and Melbourne School of Population and Global Health University of Melbourne Melbourne VIC Australia; ^30^ Department of Gastroenterology St Vincent's Hospital Melbourne VIC Australia; ^31^ Department of Medicine University of Melbourne Melbourne VIC Australia

**Keywords:** developing countries, disease elimination, hepatitis B, hepatitis C, investment case, organizational case studies

## Abstract

Viral hepatitis is a leading cause of morbidity and mortality worldwide, but has long been neglected by national and international policymakers. Recent modelling studies suggest that investing in the global elimination of viral hepatitis is feasible and cost‐effective. In 2016, all 194 member states of the World Health Organization endorsed the goal to eliminate viral hepatitis as a public health threat by 2030, but complex systemic and social realities hamper implementation efforts. This paper presents eight case studies from a diverse range of countries that have invested in responses to viral hepatitis and adopted innovative approaches to tackle their respective epidemics. Based on an investment framework developed to build a global investment case for the elimination of viral hepatitis by 2030, national activities and key enablers are highlighted that showcase the feasibility and impact of concerted hepatitis responses across a range of settings, with different levels of available resources and infrastructural development. These case studies demonstrate the utility of taking a multipronged, public health approach to: (a) evidence‐gathering and planning; (b) implementation; and (c) integration of viral hepatitis services into the Agenda for Sustainable Development. They provide models for planning, investment and implementation strategies for other countries facing similar challenges and resource constraints.

**Abbreviations**
DAAs: direct‐acting antivirals
DBS: dried blood spot
GHSSH: Global Health Sector Strategy on Viral Hepatitis
HBIg: hepatitis B immunoglobulin
HBsAg: hepatitis B surface antigen
HIV: human immunodeficiency virus
MTCT: mother‐to‐child transmission
NSP: needle and syringe program
OAT: opioid antagonist treatment
PWID: people who inject drugs
RNA: ribonucleic acid
SDG: Sustainable Development Goals
TRIPS: Trade Related Aspects of Intellectual Property Rights
WHO: World Health Organization

**Key points**

- Viral hepatitis is the 6th leading cause of death globally, surpassing all other chronic infectious diseases including HIV, tuberculosis and malaria
- Elimination of viral hepatitis as a public health threat is achievable; all WHO member countries endorsed this goal formally in 2016
- Planning, implementation and integration of national responses to viral hepatitis is ongoing, and many countries have adopted innovative approaches to address the diverse challenges of this endeavour in their local contexts
- Existing approaches demonstrate that investing in viral hepatitis is affordable and cost‐effective, provides multisectoral cost‐benefits, and alleviates the human burden of the epidemic

## 1 INTRODUCTION

Viral hepatitis contributes substantially to the global burden of disease, with 248 million people infected with hepatitis B and 71 million infected with hepatitis C worldwide.^1^ If left untreated, chronic viral hepatitis can cause life‐threatening complications, such as cirrhosis and hepatocellular carcinoma.^2^ Despite this, the public health consequences of viral hepatitis have long been neglected.^1^ In contrast to the progress in combating many other communicable diseases in recent years, viral hepatitis‐related morbidity and mortality continue to rise.^1^, ^3^ In 2010 viral hepatitis was the 10th leading cause of death, but by 2015, with 1.2 million deaths, it had overtaken HIV, malaria and tuberculosis to rise to sixth.^4^ Most viral hepatitis deaths are avertable through increased access to prevention, diagnosis and treatment.

In areas of high hepatitis B endemicity (eg Southeast Asia and sub‐Saharan Africa), perinatal mother‐to‐child transmission (MTCT) and horizontal transmission during childhood are the most common routes of infection, while sexual contacts, unsafe injecting practices, and unhygienic medical or cosmetic procedures drive transmission elsewhere.^5^, ^6^, ^7^ Risk of developing chronic hepatitis B infection is inversely related to age at infection: around 90% of infants infected perinatally develop chronic infection, unless vaccinated at birth. This risk decreases to around 30% among children infected before the age of six years and to less than 5% of persons infected as adults.^8^, ^9^, ^10^

The hepatitis C epidemic is similarly geographically diverse and mode of transmission differs substantially between regions.^11^, ^12^, ^13^, ^14^ Globally, an estimated 52% of people who inject drugs (PWID) are hepatitis C antibody positive.^15^ Lack of access to needle and syringe programmes (NSPs) and opioid antagonist treatment (OAT) result in unsafe injecting practices, which are the major route of transmission in high‐income countries.^15^, ^16^ In low‐ and middle‐income countries, additional transmission occurs in healthcare settings through substandard infection control practices.^17^

In 2016, the 69th World Health Assembly adopted the Global Health Sector Strategy on Viral Hepatitis (GHSSH) 2016‐2021. The strategy outlines five synergistic prevention and treatment service coverage targets to achieve the elimination of viral hepatitis as a public health threat by 2030 (defined as 90% reduction in incidence and 65% in mortality, see Table 1).^18^ Implementation of the strategy is expected to strengthen health systems while enabling progress toward the United Nations' Sustainable Development Goal (SDG) 3 target of universal health coverage.^19^, ^20^ Modelling studies suggest that rapid investment in diagnostic, prevention, and treatment services could achieve the World Health Organization (WHO) targets by 2030.^21^, ^22^

**Table 1:** Viral hepatitis service coverage and impact targets

Target area	Baseline 2015	2020 Target	2030 Target
Service coverage targets			
Hepatitis B virus vaccination: childhood vaccine coverage (third dose coverage)	82% of infants	90%	90%
Prevention of hepatitis B virus mother‐to‐child transmission: hepatitis B virus birth‐dose coverage or other approach to prevent mother‐to‐child transmission	38%	50%	90%
Blood safety: donations screened with quality assurance	89%	95%	100%
Injection safety: use of engineered devices	5%	50%	90%
Sterile needle/syringe set distributed per person per year for people who inject drugs	20	200	300
Viral hepatitis B and C diagnosis (coverage %)	<5% of chronic hepatitis infections diagnosed	30%	90%
Viral hepatitis B and C treatment (coverage %)	<1% receiving treatment	3 million	80% eligible treated
Impact targets			
Incidence: new cases of viral hepatitis B and C infections	Between 6 and 10 million infections are reduced to 0.9 million infections by 2030 (95% declined in hepatitis B virus infections, 80% decline in hepatitis C virus infections)	30% reduction (equivalent to 1% prevalence of HBsAg among children)	90% reduction (equivalent to 0.1% prevalence of HBsAg among children)
Mortality: viral hepatitis B and C deaths	1.4 million deaths reduced to less than 500 000 by 2030 (65% for both viral hepatitis B and C)	10% reduction	65% reduction

### 1.1 How can viral hepatitis be eliminated by 2030?

Eliminating viral hepatitis requires substantial investments in health systems strengthening and the full continuum of hepatitis services.^18^ Investing in the prevention and treatment of viral hepatitis provides many direct, indirect and cross‐sectoral economic benefits through saving lives and alleviating the cost burden of disease to the individual, their families and the state.^23^, ^24^, ^25^, ^26^ To achieve elimination at a national level, the country‐specific context and its unique challenges must be considered. A multipronged approach comprising three main pillars is most effective in addressing the local context; comprising (a) evidence‐gathering and planning the response; (b) implementation of disease‐specific activities, including investments in the delivery of care; and (c) integration of the viral hepatitis response into SDG 3 by adopting a public health approach and embedding services into universal health coverage.^27^

The necessary tools for viral hepatitis elimination are already available, but worldwide implementation of a concerted viral hepatitis response is slow and faces many challenges. These include low levels of investments in health overall; inadequate data and weak surveillance systems; poor infrastructure; low awareness among policymakers, at‐risk populations and primary care practitioners; high prices of some diagnostics and treatments; and a lack of prioritisation of viral hepatitis.^28^, ^29^ While most countries are on track to meet the WHO's 2030 target of < 0.1% Hepatitis B surface antigen (HBsAg) prevalence among 5‐year‐olds, without substantial further investments this target is currently unachievable for 20 countries, mainly in Africa and the Western Pacific. Moreover, only 12 countries are currently on track to achieve the hepatitis C elimination goal that all WHO member states adopted in 2016.^30^

**Figure 1:**
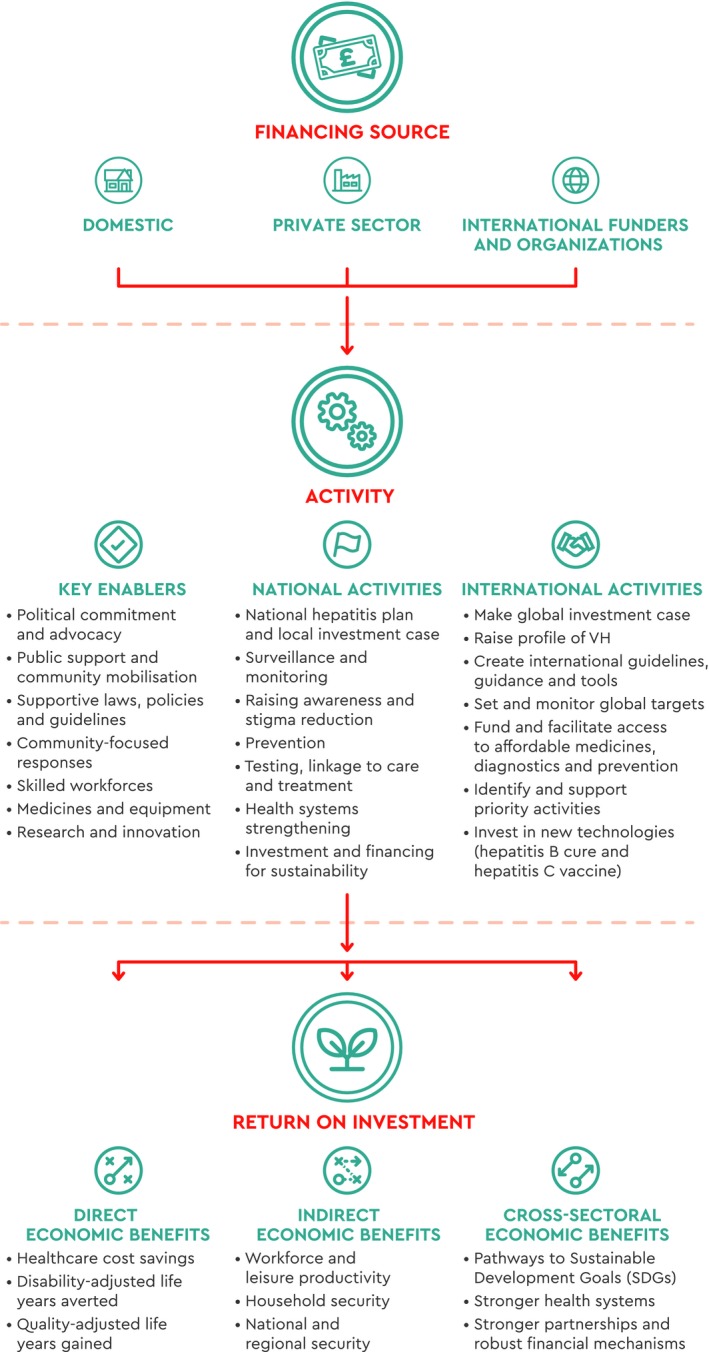
Investment framework for viral hepatitis elimination

**Figure 2:**
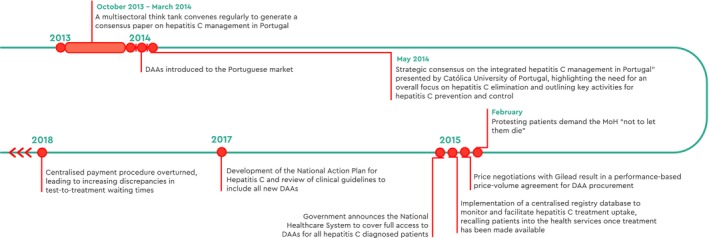
Timeline of national activities, Portugal

**Figure 3:**
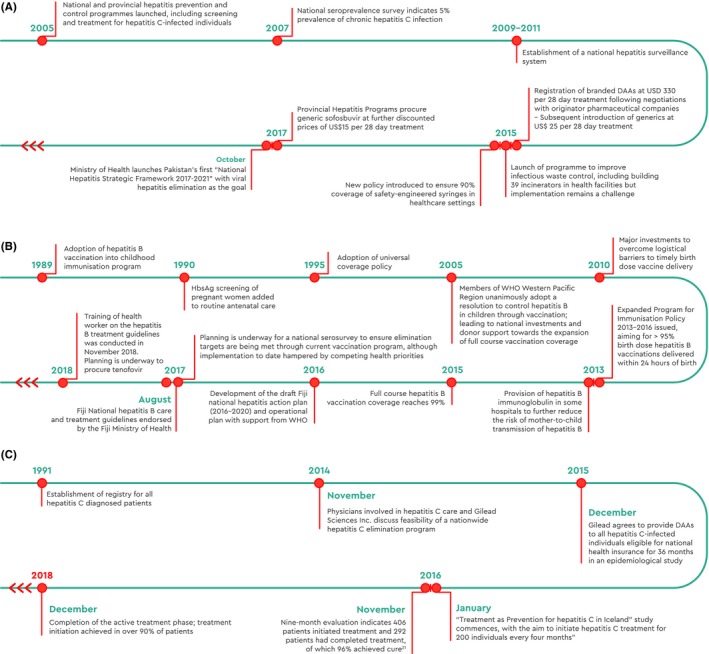
Timeline of national activities, Pakistan (A), Fiji (B), and Iceland (C)

**Figure 4:**
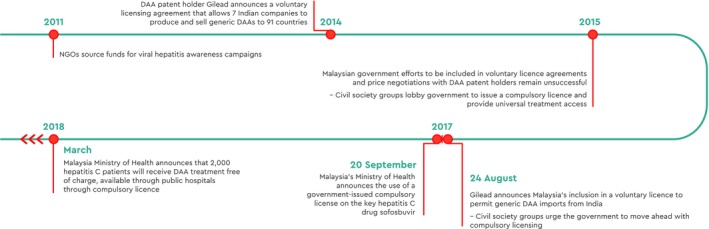
Timeline of national activities, Malaysia

We have developed a Viral Hepatitis Investment Framework outlining the resourcing required to achieve elimination, the cost of the elimination of viral hepatitis globally, and methods for countries to address existing challenges.^31^ The Viral Hepatitis Investment Framework highlights key enablers to support a comprehensive viral hepatitis response and outlines priority national and international activities to maximise return on investment (Figure 1). Using the structure of the Investment Framework, this paper presents case studies from diverse countries (Table 2) that are successfully implementing innovative strategies to eliminate viral hepatitis (see Table 3). Additional case studies listed in Table 3 are summarised in the Appendix S1 (Figures 2, 3, 4).

**Table 2:** Country characteristics

	Georgia	South Africa	Scotland	Brazil	China	Egypt	Rwanda	Australia
Population total (million, 2017)	3.72	56.72	5.3 (2011)	208.49	1.386 billion	97.55	12.21	24.6
Life expectancy at birth (years)	73	63	79	76	76	71	67	83
GNI per capita (US$)	3780	5430	42 370 (UK total)	8840	8690	3010	720	51 360
HBsAg positive population (%)	115 948 (2.64%)^2^	3.5 million (6.7%)^2^	8700 (0.2%)^2^, ^100^	1.28 million (0.65%)^2^	74.6 million (5.49%)^2^	1.34 million (1.7%)^2^	722 449 (6.7%)^2^	83 121 (0.37%)^2^
HCV‐RNA positive population (%)	150 000 (5.4%)^101^	356 000 (0.7%)^102^	37 000 (0.8%)^103^	700 000 (0.71%)^104^	9.8 million (0.7%)^105^	3.81 million (7%)^76^	175 000 (3.1%)^11^	230 000 (1%)

**Table 3:** National activities and country examples aimed at elimination of viral hepatitis

	National activities	Country examples presented in this paper
Evidence‐gathering and planning	National hepatitis plan (addressing hepatitis B, hepatitis C or both)	Georgia, Australia, Brazil, China, Egypt, Iceland, Malaysia, Portugal, Scotland, South Africa
	Accurate data to inform the response (Surveillance and Monitoring)	Scotland, Portugal, Brazil, Egypt, Georgia, Iceland, Pakistan, South Africa
	Local investment case	South Africa, Rwanda
Implementation	Raising awareness and stigma reduction	Brazil, Australia,, China, Egypt, Iceland, Malaysia, Portugal, Pakistan
	Investment in prevention	China, Fiji, Pakistan, Australia, Brazil, Iceland, Georgia, Malaysia, Portugal, Scotland
	Testing, linkage to care and treatment	Egypt, Australia, China, Georgia, Iceland, Malaysia, Portugal, Scotland, South Africa
Integration	Investment and financing for sustainability	Australia, China, Iceland, Malaysia, Rwanda
	Health Systems Strengthening	Rwanda, Brazil, Fiji, Georgia, Malaysia, South Africa

### 1.2 Evidence‐gathering and planning

Low‐quality surveillance systems and a lack of reliable cause‐specific mortality data limit countries' capacity to guide, implement and monitor effective viral hepatitis responses.^32^, ^33^ To advocate for an adequate allocation of domestic resources and to mobilise external funding support, countries should develop a national plan that sets ambitious but achievable targets, informed by a robust local investment case for viral hepatitis. Gathering accurate data to inform a targeted approach can improve the cost‐effectiveness of specific interventions.^34^, ^35^, ^36^ Since the launch of the GHSSH 2016‐2021, more countries have developed national hepatitis plans^1^ and both local and global investment cases for the elimination of viral hepatitis have been built.^31^, ^35^, ^37^ Many countries have begun collecting epidemiological data through national seroprevalence surveys or by adding key hepatitis indicators into existing surveillance systems. Below, we give examples of countries that have gathered evidence and are developing a national plan (Georgia), produced an investment case for elimination (South Africa) and obtained accurate data to inform the response (Scotland).

### 1.3 Georgia: the development of a national plan

Georgia was the first country in the WHO European region to set a hepatitis C elimination goal and develop a national plan for viral hepatitis tailored to the local context. Georgia's significant experience with HIV prevention and control programmes and the existing human and technical capacities to implement large‐scale health programmes facilitated the implementation of their national hepatitis C elimination programme.^38^ An international Technical Advisory Group assisted with describing the local hepatitis C epidemiology and proposing strategies, objectives and actions to address gaps in advocacy and awareness, surveillance, harm reduction, blood safety, infection control, and evidence‐based screening and linkage to care. Gilead Science provided direct‐acting antiviral (DAA) therapy to Georgia at no cost after the elimination programme commenced; reportedly, a key reason for their decision was the Georgian Government's commitment to an elimination response.

The programme initially focused on increasing access to affordable diagnostics; providing free DAA treatment to persons with severe liver disease at highest‐risk of hepatitis C‐related mortality; and building capacity to achieve programme goals of preventing transmission and eliminating the disease.^39^ Initial obstacles included suboptimal alignment of programme development and implementation, leading to bottlenecks in patient flow and wait lists.^40^ Training for healthcare workers was only provided after the programme launched; however, doctors have subsequently received continuous technical support.

The programme has now expanded its scope to treat every person chronically infected with hepatitis C, as outlined in the “Strategic plan for the Elimination of Hepatitis C Virus in Georgia, 2016‐2020”. Hepatitis C treatment services are provided at treatment centres located throughout the country and treatment decentralisation in harm reduction centres and primary care is ongoing. Patient out‐of‐pocket fees for diagnostics and clinical monitoring are based on ability to pay. Georgia is working to integrate its hepatitis C elimination programme into the overall health system, because this will benefit the management of other health problems such as HIV and tuberculosis.^41^ This is primarily being achieved via treatment decentralisation into primary care and harm reduction services.

**Figure 5:**
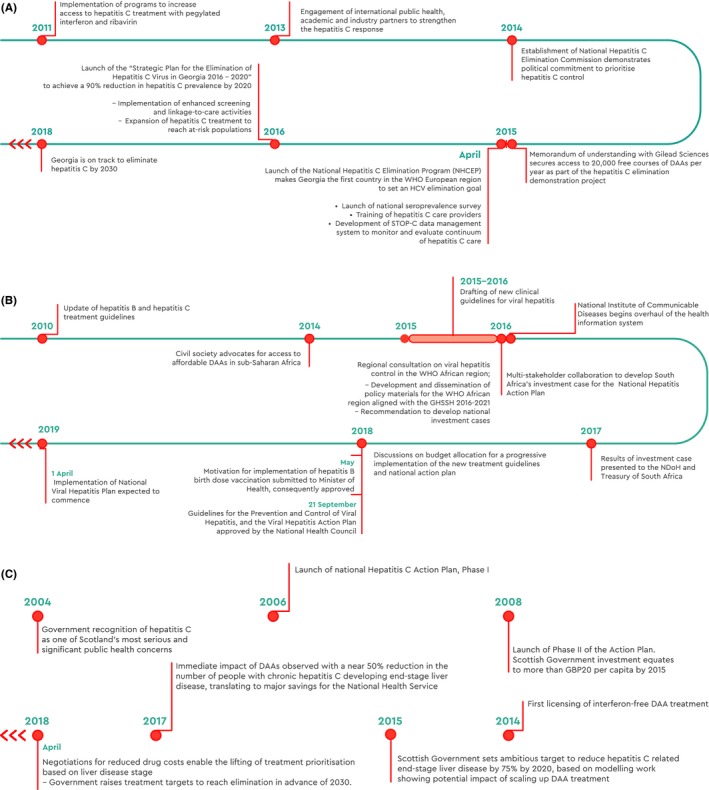
Timeline of national activities, Georgia (A), South Africa (B), and Scotland (C)

The implementation of the national action plan increased access to hepatitis C testing and linkage to care while driving improvements in monitoring and surveillance, infection control and prevention.^38^, ^41^ The evaluation of harm reduction‐based peer‐supported hepatitis C treatment demonstrated excellent treatment uptake and retention in care among PWID based in Tbilisi.^42^ By January 2019, 53 000 people had initiated treatment with the new DAAs, of whom almost 34 800 had already achieved hepatitis C cure (Figure 5A). Remaining challenges relate to the marginalised status of PWID, with stigma and discrimination preventing PWID from accessing hepatitis C services. Punitive drug laws (such as criminal responsibility for personal drug use) challenge the effectiveness of harm reduction programmes and lead to high rates of incarceration and hepatitis C transmission in prisons, where access to OST is limited. As well, as in other countries aiming for hepatitis C elimination, treatment numbers declined after the first two years of the programme, with many people being unaware of their hepatitis C status or not commencing treatment.

### 1.4 South Africa: The development of an investment case

South Africa's National Action Plan 2017‐2021 is one of the first examples of an investment case that combines tools for costing, impact modelling, cost‐effectiveness analysis, and fiscal space analysis for scaled‐up hepatitis B and hepatitis C disease control scenarios.^35^ The action plan was developed in collaboration with leading South African experts, Ministry of Health officials, and external specialists in global health policy and economics, who assessed cost and affordability, health impact and cost‐effectiveness for four priority interventions: hepatitis B birth dose vaccination, prevention of MTCT and treatment for hepatitis B and C.

The model suggests expanded hepatitis B prevention and treatment for hepatitis B and C (using DAAs for the latter) is cost‐effective and affordable in the South African context,^35^ noting that hepatitis B birth dose vaccination should be prioritised if funds are insufficient for the full implementation. The five‐year Action Plan was estimated to cost US$270 million, with the “testing, care, and treatment” component being the most costly. Whilst this is a significant amount of money, seen against 5‐year HIV expenditure, the cost of the Hepatitis Action Plan is estimated to be less than 4% of the projected HIV spend in South Africa.^43^ Integrating the action plan into the existing health system, particularly maternal and child health and HIV/AIDS services, was estimated to improve implementation feasibility.

The modelling data suggest the initial five‐year investment could avert an estimated 13 000 hepatitis B‐related deaths and 7000 hepatitis C‐related deaths. Moreover, a continued expansion of the treatment programme beyond 2021 has the potential to avert 672 000 hepatitis B‐infections and 60 000 deaths averted from hepatitis C‐related liver disease, which would put South Africa firmly on the path to achieve elimination by 2030 (Figure 5B).^35^

The multi‐stakeholder approach used to develop an investment case for the cost‐effectiveness and affordability of hepatitis control and elimination for South Africa provides a template for other countries.^44^ Implementation of the investment case‐informed Viral Hepatitis Action Plan is expected to commence on 1st April 2019, with five priority interventions during the first year: (a) hepatitis B birth dose vaccination; (b) healthcare worker hepatitis screening, vaccination and training in viral hepatitis (c) increasing awareness, diagnosis and management of Hepatitis B virus (Tenofovir is on the Essential Medicine list); (d) registration of DAAs and price negotiations; (e) a comprehensive package of viral hepatitis services for key populations – men who have sex with men and people who use/inject drugs.

Key obstacles to the response are a lack of funding being allocated to the Programme due to fiscal constraints; a shortage of trained health workers; lack of knowledge about viral hepatitis in the general public; viral hepatitis‐related stigma; limited access to harm reduction services; and punitive drug laws. There is a need to improve viral hepatitis services in other key populations, including prisoners, sex workers and men who have sex with men. Moreover, DAAs are yet to be registered in South Africa due to administrative delays at the South African Health Products Regulatory Authority, preventing broader hepatitis C treatment scale‐up.

In order to address these obstacles, the South African Viral Hepatitis Working Group has established three subcommittees to oversee implementation of the hepatitis B birth dose vaccine, training of healthcare workers in conjunction with training on new HIV treatment regimens, and hepatitis C micro‐elimination programmes.

### 1.5 Scotland: accurate data to inform the response

In Scotland, advocates used political pressure and scientific evidence to raise awareness of the human impact of hepatitis C and its links to inequalities, which generated political consensus to support significant funding and evidence‐based policy initiatives.^45^ Social and political recognition of the scale of the problem galvanised policymakers into action. Innovative strategies such as the introduction of dried blood spot (DBS) sampling in community drug services made the model of viral hepatitis care more acceptable to affected communities and helped overcome barriers to testing.^46^ Adopting a project management approach ensured achievable goal‐setting and controlled ongoing cost. Substantial investment in a robust monitoring and surveillance system – combined with ambitious treatment targets – facilitated progress and demonstrated immediate impact, which helped to sustain momentum.^47^ Scotland's response – the National Hepatitis C Action Plan – has been a phased one. Launched in 2006, Phase I focused on gathering evidence to inform and generate proposals for the development of hepatitis C services and identify the additional investment required. Subsequently, in Phase II the Scottish Government committed funds to substantially improve prevention (including increasing coverage of harm reduction services), diagnosis and treatment services and deliver evidence‐based actions throughout the country for improved hepatitis C prevention and control (Figure 5C). Since 2011, the Hepatitis C Action Plan has been integrated with other national policies within the Scottish Government's Framework on Sexual Health and Blood Borne Viruses, which adopts a multi‐agency outcomes‐based approach with a strong focus on challenging inequalities.^48^, ^49^

The national strategy to improve prevention, diagnosis and treatment services led to a significant decline in hepatitis C incidence, more new diagnoses, more people undergoing hepatitis C treatment and achieving cure, reductions in liver‐related morbidity and mortality, and a decreased population prevalence of chronic hepatitis C.^47^, ^50^, ^51^, ^52^ Scotland's example showcases the utility of evidence‐based national hepatitis C strategies in reducing the financial and societal burden of the epidemic^52^, ^53^ and provides a working model for other countries to follow.

Despite the progress made in improving harm reduction services in Scotland during the era of interferon‐based treatment, the prevalence of hepatitis C infection had remained stubbornly high. The recent scale‐up of DAA therapy to PWID is hoped to bring a treatment‐as‐prevention benefit.^54^ While the roll‐out of DBS testing was effective at diagnosing infection, a substantial minority of the infected population remains undiagnosed. It has proven difficult to fully engage general practitioners in case‐finding initiatives, with awareness‐raising campaigns having limited success.^55^, ^56^ However, it is hoped that the availability of DAAs within primary care and other community settings will increase treatment uptake as the utility of the new therapies is recognised.

### 1.6 Implementation

Globally, nine of the 10 people living with viral hepatitis are unaware of their infection,^33^ and lack of public knowledge is often compounded by viral hepatitis‐related stigma and discrimination. Implementation of a viral hepatitis strategy should therefore include awareness‐raising activities to generate demand for viral hepatitis care (eg through social media campaigns, such as in Brazil^57^) in conjunction with supportive laws, policy and guidelines that aim to reduce stigma and enable the establishment of community‐focused responses.^46^

Prevention activities should be implemented and scaled up to effectively eliminate viral hepatitis transmission. A highly effective hepatitis B vaccine has been available since the 1980s, and early immunisation plus the distribution of hepatitis B immunoglobulin (HBIg) to at‐risk infants prevents perinatal transmission, as China has demonstrated.^58^ Harm reduction interventions, including NSPs and provision of OAT, cost‐effectively reduce primary and reinfection incidence among PWID.^59^, ^60^, ^61^ Iatrogenic transmission can be eliminated through routine screening of blood supply^62^ and implementation of safe infection practices (including reducing unnecessary injections, staff training and effective waste management),^63^ while simultaneously contributing to health systems strengthening.^4^, ^64^

Finally, implementation of a viral hepatitis response must aim to optimise the viral hepatitis care cascade by substantially improving testing rates, linkage to care and treatment numbers. The case of Egypt (and Iceland, see Appendix S1) demonstrates that concerted efforts enable substantial advances towards the WHO targets of 90% of people diagnosed and 80% of eligible people treated.^30^, ^65^, ^66^

Below are examples of implementation: raising awareness and stigma reduction (Brazil), investment in prevention (China), and investment in testing, linkage to care and treatment (Egypt).

### 1.7 Brazil: raising awareness and stigma reduction

Brazil, a middle‐income country, has been providing universal access to antiretroviral therapy for HIV since 1996, driven by strong political will, multisectoral mobilisation and use of Trade Related Aspects of Intellectual Property Rights (TRIPS) flexibilities, and civil society engagement.^67^ It has championed the cause of viral hepatitis and advocated for an intensified global response for many years. Learning from its successes in reversing the trend of the HIV epidemic, Brazil established a national hepatitis programme informed by up‐to‐date estimates of disease prevalence, international guidelines and cost‐effectiveness in the Brazilian Unified Health System.^57^ Brazil invested in universal hepatitis B vaccination, increased capacity for hepatitis C testing in HIV services, expanded its laboratory network and set up a referral system for hepatitis patients. To reach the target population, the Ministry of Health conducted new public awareness and diagnosis campaigns using a variety of media with endorsement from civil society and the scientific community.^57^

Brazil was able to obtain an unprecedented discount for an upper‐middle‐income country through price negotiations with originator pharmaceutical companies. Between 2015 and 2018 it provided treatment to nearly 90 000 people, and is expected to treat another 50 000 patients in 2019, largely thanks to the strong advocacy of civil society.

**Figure 6:**
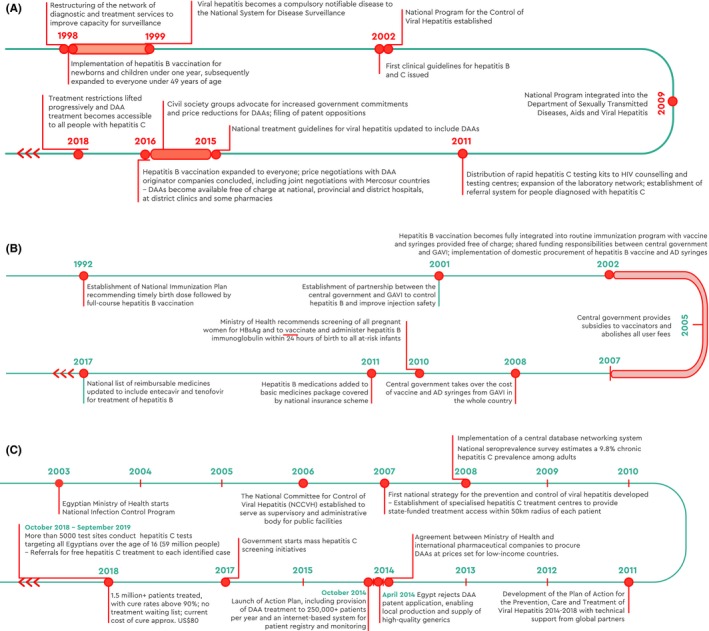
Timeline of national activities, Brazil (A), China (B), and Egypt (C)

The remarkable process applied in Brazil was based on epidemiological data and scientific evidence, and motivated by its engagement with the SDGs, which may inspire other countries to identify ways to achieve these goals by 2030.^57^ Brazil has pledged to provide free hepatitis C treatment to everyone infected and is one of 12 countries on track to achieve hepatitis C elimination by 2030 (Figure 6A).^30^

Despite this progress, geographical, social and economic disparities in Brazil challenge the provision of equitable service access across varied geographical regions. Brazil is working to improve diagnosis rates and mitigate losses to follow‐up, resulting from the long delays between diagnosis and treatment initiation arising from small numbers of specialists who can provide DAA treatment.^68^

### 1.8 China: investment in prevention

China is home to nearly one third of all people living with hepatitis B infection globally. HBsAg prevalence is estimated at 5.5%^2^ and hepatitis B causes over 300 000 deaths annually due to liver diseases.^69^ The implementation of a universal hepatitis B vaccination programme for infants has reduced chronic hepatitis B incidence dramatically during the past two decades. The full implementation of a national programme for the prevention of MTCT guarantees adequate supply of HBIg for at‐risk newborns. Domestic procurement of the hepatitis B vaccine and auto‐disable syringes ensures sustainable supply chains and stimulates regional industry and technology markets.^70^

Driven by strong political commitment and with support from the Global Alliance on Vaccine and Immunization, including an investment of ~ USD76 million to subsidise the hepatitis B catch‐up vaccination programme for 15 million children through public‐private partnerships such as with Rotary and the ZeShan Foundation,^71^ multiple strategies were developed and implemented collaboratively (Figure 6B). As a result, >95% of infants receive the hepatitis B vaccine within 24 hours of birth.^72^, ^73^, ^74^, ^75^ This programme led to a nationwide catch‐up vaccination drive for children up to the age of 15, reaching 68 million people over a 3‐year period (2008‐2011) (private communication). Between 1992 and 2013, China's efforts have prevented 90 million cases of chronic hepatitis B infection and 24 million fewer people are carriers of the virus –a massive reduction in the global burden of viral hepatitis.^70^

Although China has made considerable progress with hepatitis B, systemic obstacles to the elimination of MTCT remain. The physician‐centred approach of the medical service infrastructure discourages affected pregnant women from seeking timely treatment, because physicians trained to provide treatment (ie obstetricians, gynaecologists, gastroenterologists and infectious disease specialists working in central hospitals) are often reluctant to do so. Moreover, China is yet to implement a comprehensive national strategy addressing its hepatitis C epidemic. Few DAAs have been approved and their high cost restricts inclusion in basic health insurance programmes; consequently, DAA treatment is not universally available. Policy changes and education campaigns are needed to overcome stigma and discrimination and improve diagnosis rates, and linkage to care needs improvement.

### 1.9 Egypt: testing, linkage to care and treatment

Egypt has a very high burden of hepatitis C infection and disease, with approximately 7% of Egyptians aged 18‐59 living with chronic hepatitis C infection in 2015.^76^ This large reservoir of active infection and continued unsafe medical practices contribute to ongoing transmission; in 2016, an estimated 150 000 Egyptians were newly infected.^77^

Egypt is committed to ending its generalised hepatitis C epidemic. It has developed one of the largest national programmes for hepatitis C treatment.^78^ Egypt provides free and universal access to locally produced DAA treatment, as part of a national action plan for the prevention and control of viral hepatitis. To maximise efficiencies, the country has rolled out mass screening since October 2018, providing direct linkage to hepatitis C care. Over six months, more than 49 million people were reached, of whom over 2 million were diagnosed as hepatitis C‐antibody positive (in addition, >2.5 million possible cases of diabetes and > 10 million possible cases of hypertension were identified and referred for further assessment and management). Of hepatitis C patients linked to care and confirmed as ribonucleic acid (RNA) positive, 750 000 started treatment.

By 2019, over 2.4 million Egyptians had been treated, and the country is on track to achieve WHO elimination targets in spite of its high hepatitis C prevalence (Figure 6C).^78^, ^79^ Egypt's response was facilitated by strong political will and government advocacy, effective price negotiations, removal of patent barriers on DAAs and ability to produce DAAs locally.^66^, ^80^

Despite great progress Egypt's response is challenged by difficulties in capturing non‐responders to treatment and lack of appropriate medications to initiate retreatment. Moreover, children under 12 years old cannot be treated because the medications have not yet been approved for this age group. Finally, plans and strategies for surveillance to reliably capture whether hepatitis C elimination targets have been met are not fully developed.

### 1.10 Integration

The cost burden of viral hepatitis diagnostic tests and treatment – in particular the new DAA treatment for hepatitis C – challenges the feasibility and sustainability of effective viral hepatitis elimination activities. Unlike for other major communicable diseases such as HIV, tuberculosis and malaria, there is little funding available for viral hepatitis at an international level and most countries lack dedicated hepatitis budgets or programmes.^18^ Although the private sector (such as pharmaceutical companies) and international funders and organisations are important actors in global elimination efforts, most funding will have to be mobilised from public, domestic sources to ensure the sustainability of viral hepatitis services as part of a broader effort to increase overall investments in health.^29^, ^81^, ^82^ Increasing investment in infrastructure and health service delivery (ie health systems strengthening) is not only a key enabler for viral hepatitis elimination but a requirement to reach the overarching SDG 3 for health and its target of universal health coverage.^19^ Ensuring that hepatitis services are integrated within these systems can reduce costs, compared to an isolated, non‐strategic approach,^31^ exemplified here in the case of Rwanda.

Integrating the viral response into the health system by utilising existing structures and trained workforces can save costs and generate efficiencies, as well as maximising access to services for key risk populations.^83^ For example conducting viral hepatitis testing at HIV services is likely to yield high diagnosis rates because people living with HIV have a higher risk of hepatitis B or hepatitis C co‐infection, and may improve their engagement in care.^84^ However, it is important to look beyond integrating the response into HIV programmes, because further opportunities exist to broaden the viral hepatitis response by integrating it within tuberculosis, maternal and child health, and diabetes programmes. Also such an approach may not be useful in countries with generalised epidemics (such as China and Egypt) that require population‐based approaches to testing and treatment.

Even when the response is integrated within the broader health system, there will be extra costs due to the need to expand services and to increase staffing levels to accommodate the increased activity. For example, additional time is needed to administer a hepatitis B vaccine or to provide post‐test counselling for positive test results.^82^ Due to concerns about extra costs and workload, efforts to integrate viral hepatitis responses into existing systems and platforms may receive substantial pushback, particularly initially. However, there is no evidence to support the notion that introducing viral hepatitis care into these systems causes existing structures to collapse.^85^

Moreover, multiple countries have been able to make treatment accessible to the broader population by successfully negotiating with patent holders (eg Australia), making use of patent licenses either available directly from the patent owner or those held by the Medicines Patent Pool (eg Rwanda),^86^ or using TRIPS flexibilities to circumvent patent barriers to accessing lower priced generic DAAs (eg Malaysia, see Table 4 and Appendix S1).^87^

**Table 4:** Hepatitis B vaccination coverage and procurement status of hepatitis C medicines

Country	Hepatitis B vaccination coverage (2019)^75^		Hepatitis C treatment procurement (2017)^107^			
	Three‐dose vaccination <1 y	Timely birth dose	DAAs registered in country	Voluntary license (VL) or Compulsory/government‐use license (CL)	Generic local production	Support from originator company
Australia	94%	91%	Yes	—	No	No
Brazil	86%	76%	Yes	No	No	No
China	99%	96%	Yes	No	No	No
Egypt	95%	13%	Yes	VL	Yes	Yes
Fiji	93%	95%	No	VL	No	No
Georgia	92%	94%	Yes	VL	No	Yes
Iceland	a	a	Yes	—	No	Yes
Malaysia	98%	88%	Yes	CL and VL	No	No
Pakistan	86%	<1%	Yes	VL	Yes	Yes
Portugal	98%	97%	Yes	—	No	No
Rwanda	98%	0%	Yes	VL	No	Yes
Scotland	<1% (UK)	<1% (UK)	Yes	—	No	No
South Africa	74%^2^	n/a	Yes	VL	No	No

a Estimates of hepatitis B vaccination coverage were produced only for countries with universal birth dose policy.^75^

Below are examples of integration: health systems strengthening (Rwanda) and investment and financing for sustainability (Australia). Importantly, the health systems in both countries have coped with this considerable scale‐up of treatment and care.

### 1.11 Rwanda: expanding on universal health coverage

Rwanda is a low‐income country that is using a public health framework for hepatitis control and care to progress on its aim to achieve universal health coverage.

The country has made tremendous gains in maternal and child health, malaria, tuberculosis and HIV outcomes. The Rwandan Government now invests major resources in viral hepatitis, using programmatic steps that form a blueprint for other low‐income countries in the region.^88^ Key elements of Rwanda's programme for viral hepatitis prevention and treatment include:

- Simplified treatment algorithms not requiring hepatitis C genotype or hepatitis B viral load and largely able to be delivered by nurses at health centre level
- Selective partnerships and preferred suppliers to drive down price, consolidate the supply chain and streamline diagnostic platforms to avoid siloed approaches to healthcare^89^
- Study of necessary resources for efficient implementation
- Development of a training programme for health staff
- Development, funding and implementation of birth dose vaccination for hepatitis B.

**Figure 7:**
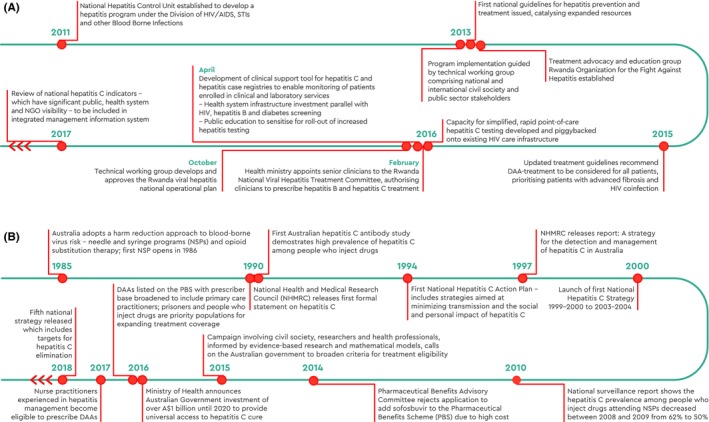
Timeline of national activities, Rwanda (A) and Australia (B)

To ascertain feasibility and ensure financing for sustainability, a national operational plan was developed to demonstrate priority‐setting of key activities and provide costing estimates for different levels of coverage of screening, diagnosis, and treatment of both hepatitis B and C.^88^ Several initiatives were used to secure funding, including support from international donors, in particular the Clinton Health Access Initiative. Rwanda has a voluntary licensing agreement for DAAs and is therefore able to produce medication at reduced cost (approx. US$ 560 in 2017).^66^, ^88^ Rwanda's Essential Medicines List includes generic hepatitis B medicines treatment; this is subsidised by government for people with HIV coinfection. All major private health insurance companies (as well as military medical insurance) reimburse for the cost of DAAs, and the Rwanda social security board covers 85% of the cost. Ultimately, the aim is to provide reimbursement for hepatitis C diagnostics and treatment by the community‐based health insurance scheme.^88^ As of June 2017, 2500 patients had been treated with DAAs and treatment for 9000 additional patients had been procured (Figure 7A). Rwanda aims to establish treatment capacity at all 48 district hospitals countrywide by 2020.

Major ongoing barriers to addressing viral hepatitis in Rwanda include the lower awareness of, and priority given to, viral hepatitis compared to other infectious diseases (eg malaria and HIV) and the competing priorities for limited public‐sector health funding. A prior strategy (from 2011) that failed and has since been abandoned was to develop local viral hepatitis treatment guidelines based upon international consensus guidelines, without sufficient attention to the resources required for implementation (including particularly laboratory testing and availability of medications) or the skills and experience required of clinicians. These guidelines thus lacked local contextualisation and recommended unavailable or unaffordable management; consequently, they were impractical and did not influence daily clinical practice greatly.

### 1.12 Australia: a multipronged approach to elimination

In 1999, Australia was one of the first countries to implement and subsequently refine their national hepatitis C strategies and has since then become a best practice model for hepatitis C elimination. Key to Australia's response, including achieving universal treatment access (described below), has been strong community advocacy, health research, health sector and political leadership that foster continued commitment to the WHO 2030 elimination targets, including a timely response to new challenges.^90^ Australia has had a long and sustained harm reduction approach to injecting drug use, with engagement of civil society, the health sector and government. This is beneficial in reducing bloodborne virus transmission and cultivates a point of engagement with PWID in providing health and social services.^59^, ^60^, ^61^, ^91^ Strong engagement with PWID is crucial to Australia's response.

By negotiating a volume‐based, risk‐sharing agreement with originator pharmaceutical companies and committing over AUD1 billion to the purchase of DAAs between March 2016 and February 2021, Australia obtained major discounts on drug list prices and as a consequence limited its expenditure. With no cap on treatment numbers,^92^ there is an incentive to diagnose and treat as many people as possible to maximise Australia's investment and its public health benefits. This provides an enabling environment to prioritise high‐prevalence groups with ongoing risks for treatment, such as PWID and prisoners, necessary to achieve hepatitis C elimination. In addition, all registered medical practitioners are able to prescribe DAA therapy, enabling more convenient, patient‐centred care. In Australia, close collaboration between people living with hepatitis C, community organisations, clinicians and policymakers facilitated improved access to diagnosis and treatment scale‐up (Figure 7B).

Australia aims to treat around 15 000 to 20 000 hepatitis C patients per year, to reach the WHO target to eliminate viral hepatitis as a major public health threat by 2030. This early commitment to achieving elimination and provide unrestricted treatment access enabled rapid treatment uptake during the first two years of DAA availability.^90^ Between March 2016 and late 2018, over 70 000 patients (around 30% of all infected Australians) were treated. The proportion of individuals prescribed DAA treatment by general practitioners increased from 8% in March 2016 to 39% in June 2017.^93^ With the successful implementation of its hepatitis C strategy – a global benchmark for best practice^94^ – Australia is on track to achieve elimination by 2030.^95^

Of concern in Australia is the continuing drop off in the number of patients undergoing screening and confirmatory testing and treatment since March 2016.^93^ While treatment numbers have been sufficient to maintain the elimination targets, further decline could put the elimination effort at risk. The decline in treatment numbers demonstrates that universal availability of DAA treatment alone is not enough to improve access to diagnosis and retention in care. Continued political commitment and policy and health system interventions are needed to facilitate treatment access for key populations to sustain momentum and overcome ongoing programme challenges to treatment scale‐up.

## 2 DISCUSSION

The broader benefits of investing in the elimination of viral hepatitis – including progressing on the SDGs ‐ are increasingly being recognised. Countries with different income levels, public health infrastructures and policy environments are effectively responding to their respective epidemics.

Attaining the viral hepatitis elimination targets set by the global community in 2016 is achievable but also highly ambitious and comes with considerable challenges (see Appendix S1). These should not be ignored, but instead considered and addressed both at a global level and within the local context to invigorate and maintain national elimination efforts. Gathering sufficient funds to finance viral hepatitis programmes continues to be difficult among competing health priorities and budget constraints. Not all countries currently benefit from generic competition, with heavily burdened middle‐income countries (eg China, Malaysia, Thailand) struggling to afford higher drug prices. A further obstacle is the increasing cost of diagnostics; for example, in Egypt expenditure on diagnostics now exceeds that on hepatitis C treatment.^68^ There are few WHO prequalified point‐of‐care viral hepatitis tests and little production of low‐cost high‐quality generic tests. In many low‐income countries, strengthening primary health care systems for maternal and child health, developing laboratory capacity, and improving weak registration and procurement systems for essential medicines is an ongoing challenge. For hepatitis B, cold chain barriers to vaccination including birth dose delivery exist, and while the controlled temperature chain presents a cost‐effective alternative that could vastly improve coverage^96^ it is yet to be broadly adopted.

Even in countries such as Australia, where there is close collaboration between community, government and health practitioners to guide implementation, elimination cannot be guaranteed because many patients remain undiagnosed and/or do not access treatment.^93^, ^97^, ^98^ Identification of sufficient numbers of infected patients needing treatment remains a challenge globally; meanwhile, in countries where scale‐up of a viral hepatitis response is pending, demand for viral hepatitis testing and treatment can outstrip available testing and treatment facilities,^85^ creating bottlenecks within the care cascade leading to losses to follow‐up. High levels of stigma, discrimination, social marginalisation and legal impediments imposed on key populations at risk or infected with viral hepatitis (eg PWID, prisoners, men who have sex with men, sex workers) is a major issue preventing engagement in care and service access^84^ and in many countries legal protections remain insufficient.^68^ The impact of regressive policies and laws on the elimination response cannot be underestimated.

The country case studies presented here demonstrate that major gains are possible in spite of these challenges – across various epidemic profiles, within a diverse range of resource constraints and within relatively short‐time frames. The case studies illustrate that political will and commitment, civil society advocacy, donor support and community acceptance are crucial and can make a difference. From concerted screening efforts in Egypt and using innovative approaches to increase hepatitis C testing in Scotland, to building local investment cases in South Africa, to integrating viral hepatitis services into existing health infrastructure in Brazil and Rwanda, these pioneers provide important models for other countries to follow. In all countries multi‐stakeholder engagement of national and international experts, civil society organisations and affected communities form critical components across the three pillars of evidence‐gathering and planning, implementation and integration.

On a global level, civil society bodies such as the World Hepatitis Alliance are instrumental in generating pressure on governments and international organisations, providing an evidence‐based approach to the response.^82^ Locally, robust evidence and civil society advocacy helped to achieve political commitment and facilitated the development of national plans. Collaboration and cooperation between civil society, the pharmaceutical industry and government smoothed the introduction of prevention and control programmes. Such unified, evidence‐informed strategies at the political and technical levels are crucial to attract and sustain commitment and financing. Learnings from these country examples and other local projects demonstrating the feasibility of elimination (eg micro‐elimination projects^36^, ^99^) can help persuade policymakers in other countries to support viral hepatitis prevention and control plans. In‐country and global advocacy must be maintained to keep viral hepatitis high on the political agenda.^82^

## 3 CONCLUSION

At the 2016 World Health Assembly, the global community uniformly endorsed the unique opportunity to eliminate viral hepatitis as a public health threat. Although an ambitious goal, technological advancements have made it scientifically feasible and increasing recognition of the public health threat posed by viral hepatitis provides the grounds for substantial political and societal support. The broader benefits of investing in the elimination of viral hepatitis – including progress on the Agenda for Sustainable Development – are now well recognised. Sustaining political momentum will be critical if elimination efforts are to be successful and more countries will need to take action if global elimination of viral hepatitis is to be achieved. Looking to existing approaches that address viral hepatitis can facilitate political support, because they demonstrate that investing in viral hepatitis is cost‐effective and can be made affordable, provide multiple economic benefits, and above all alleviate the human burden of the epidemic. The case studies presented in this paper provide clear and feasible examples of successful approaches taken by low‐, middle‐ and high‐income countries with diverse epidemics of hepatitis B and C to achieve the WHO 2030 viral hepatitis elimination targets.

## CONFLICT OF INTEREST

AHS reports grants and travel funding to her institution from ViiV Healthcare. ETH is the former director of the Medicines Patent Pool. NS has received investigator‐initiated research funding from Gilead Sciences. JVL reports grants and personal fees from AbbVie, Gilead Sciences and MSD, personal fees from CEPHEID and Janssen outside the submitted work. MES is principal investigator in an investigator‐initiated trial sponsored by Gilead Sciences (received no PI fees, trial closed April 17th 2019) and reports an educational grant to travel to EASL 2019 (Gilead Sciences). SJH received honoraria from Gilead, unrelated to submitted work. MH's institute receives investigator‐initiated research funding from Gilead Sciences, Abbvie and BMS. JH received the Gilead Sciences Australia fellowship (2017). DW, CK, RA, RBL, MB, LA, AG, SH, RH, WL, RBM, SO, RP, MS, CWS, TS, MT, TW and ESS have nothing to declare.
