# Supplementary material for: Innovative strategies for the elimination of viral hepatitis at a national level: A country case series
Source: Liver Int. 2019 Sep 4;39(10):1818–36. doi: 10.1111/liv.14222 (PMC6790606; doi:10.1111/liv.14222)
Supplement: Supplementary file 1 [file LIV-39-1818-s001.docx]

# Appendix 1: Supplementary material

This appendix presents additional case studies of countries effectively addressing viral hepatitis in their local context (also summarised in Table 5). A summary table provides an overview of remaining challenges and how political commitment has been achieved in each country (Table 6).

## Evidence-gathering and planning

## Portugal: Evidence-based advocacy enables impactful hepatitis C response

Portugal used evidence-based, data-driven and decision-oriented policy making to tackle low investments in public health, high numbers of undiagnosed patients, and outdated hepatitis C care guidelines.^1^

The country negotiated a volume-based agreement with the DAA originator company, Gilead Science Inc.; the first value-based/risk-sharing agreement in which pharma companies were paid per cure instead of paying per pill or per cycle of treatment. The full negotiation and payment procedures were centralized by the government, freeing up hospital boards to focus on treating as many patients as possible (Figure 5).^[[1]](#footnote-1)^ All new DAAs are now available in Portugal lowering overall cost, such that DAA prices are no longer a limiting factor to the Portuguese hepatitis C response.

As of February 2019, over 22,400 patients have been diagnosed with chronic hepatitis C in the national hepatitis C registry and their treatment has been authorised; nearly 21,000 treatments were initiated and 11,938 patients had achieved cure. It was estimated that implementing the National Action Plan would reduce the lifelong healthcare cost by >270 million euros in total, if 13,000 patients achieved cure.^2^

Preliminary impact evaluations suggest a 73% reduction in the incidence of hepatocellular carcinoma; a 92.5% reduction in the need for liver transplants due to hepatitis C; and a 93.2% reduction in the development of cirrhosis.^3^

The Portuguese case has shown the power of policy change as a source of blood-borne infection control. Decriminalization of drug consumption had a direct positive impact on incidence rate of HIV and viral hepatitis. Integrating hepatitis care into existing HIV programs has also avoided creation of new infrastructure.

As only approximately one-third of all people estimated to be living with hepatitis C have been diagnosed, challenges remain. Despite having universal access to treatment mathematical modelling has demonstrated that further policy changes are required for Portugal to be able to achieve hepatitis C elimination by 2030, including expansion of DAA treatment in prison settings.^4^

## Implementation

## Pakistan: Policy for prevention of iatrogenic transmission

Pakistan is a lower-middle income country that harbours 10% of the global hepatitis C burden.^5^

Risk factors for hepatitis C transmission in Pakistan are primarily health system related; including unnecessary injections and poor infection control practices in the healthcare setting.^6^ Few hepatitis C-infected individuals know their status,^7^ calling for a substantial upscale of testing and linkage to care.

Significant political commitment to address this public health concern enabled the launch of the National Hepatitis Strategic Framework 2017-21 in Pakistan (Figure 6.a). New policies target iatrogenic transmission. Automatic screening of all patients in contact with the hospital system improves hepatitis C diagnosis rates.^5^ Following negotiations between the National Technical Advisory Group of hepatitis experts and originator pharmaceutical companies, new DAAs were registered in Pakistan at a 98% discount from the US market price. Subsequently, generic competition has resulted in DAA prices of US$ 40-50 per 12-week course – to date the lowest price worldwide.^7, 8^ This has set the stage for scale-up of treatment by the public sector. Pakistan now provides free diagnosis, treatment and care to hepatitis patients in all provinces, through four Hepatitis Prevention and Control programmes.^9^ Modelling data suggests at least 880 000 annual DAA treatments are required to achieve hepatitis C elimination by 2030. Targeting treatment towards persons with cirrhosis and people who inject drugs (PWID) would reduce this number to 525 000 annual treatments, based on the premise that prevention activities halve current transmission risks.^5^

Implementation is challenging and the scale of treatment delivery differs between provinces. With 90,000 patients on DAA treatment in 2018, the province of Punjab is currently spearheading elimination efforts. More recently significant efforts to scale up treatment have commenced in other provinces such as the Sindh which is implementing an initiative to treat 7000 individuals in high prevalence districts.

## Fiji: Addressing viral hepatitis through related health platforms

Fiji is part of the Pacific Islands and Territories and comprises more than 100 inhabited islands. The country has moderate-high prevalence of hepatitis B, with 4.8% of its population chronically infected. In the WHO Western Pacific Region to which Fiji belongs, hepatitis B-related mortality is greater than for tuberculosis, HIV infection and malaria combined.^10, 11^

Fiji offers government subsidised testing and blood donation screening for hepatitis B. The country invested substantially in antenatal care and improvements in healthcare access to ensure virtually 99% of the Fijian population deliver their babies within a health facility or with a skilled birth attendant where hospital access is not possible.^12^ These investments have not only improved antenatal care and maternal and child outcomes, but provided the vehicle for successful delivery of the hepatitis B vaccine to all newborns. Despite limited health infrastructure, diverse geographical populations and no GAVI sponsorship of national vaccination programs in Fiji, hepatitis B birth dose vaccination coverage increased to 98% in 2014 resulting in substantial reductions in the HBsAg prevalence among children under 5 years; from 5-10% prior to 1995 to achieving the WHO 2017 milestone of <1% prevalence (Figure 6.b).^13, 14^

Unfortunately, resources are restricted and insufficient to implement a broader range of interventions. Infrastructural challenges, including geographical isolation; limited laboratory capacity at divisional and sub-divisional level and lack of skilled medical staff further inhibit Fiji’s viral hepatitis response. Fiji is yet to develop a comprehensive national strategy which includes estimates of the economic burden of viral hepatitis and implementation.

## Iceland: Eliminating hepatitis C from a small island country

Iceland is a high-income country whose population of 340,000 is covered by national health insurance. Approximately 1,100 Icelanders are chronically infected with hepatitis C, the majority with a history of injecting drug use.^15^

In 2016, Iceland launched a nationwide hepatitis C elimination program offering universal DAA therapy. Aiming to treat the majority of patients within the first two years of the program, the initial focus was on reaching PWID, prisoners and those at highest risk of liver cirrhosis. Rapid point-of-care testing for hepatitis C and HIV facilitates access to hepatitis diagnostics and treatment for hard-to-reach populations. To further stimulate uptake of hepatitis C care, testing and treatment activities are complemented by tailored public awareness campaigns in the mass media.

This multipronged approach, combining improved harm reduction strategies, scale-up of prevention, testing and early treatment of hepatitis C in hospital and community settings puts Iceland on track to achieving hepatitis C elimination well in advance of the 2030 WHO elimination targets (Figure 6.c).^16^

During the first three years of the program treatment has been initiated in >95% of patients with known infection and an estimated 90% of the total infected population. However, despite the high overall success there is a small group of people who remain difficult to engage in care, mostly people who are actively injecting drugs. Rising numbers of PWID and increasing drug use are challenges to elimination which require a targeted response. Visitors from abroad, asylum seekers and immigrants with pre-existing hepatitis C infection continue to contribute to hepatitis C prevalence in Iceland. However, these patients are usually not at risk of transmitting hepatitis C to the broader community.

## Integration

## Malaysia: Affordable medicines through TRIPS flexibilities

Malaysia made a commitment to address its high hepatitis C burden and achieve the WHO elimination targets, requiring a drastic scale-up in both hepatitis C screening and treatment.^17^ An initial exclusion from Gilead’s voluntary licensing agreement, which prioritises low- and middle-income countries with greatest disease burden^18-20^ impeded Malaysia capacity for treatment scale-up. A 12-week course of treatment with sofosbuvir cost nearly half the average annual household income in Malaysia (US$ 12,000)^21^, putting it well out of reach of most patients. In response, Malaysian NGOs lobbied the government to issue a compulsory licence and expand access to effective DAA therapy in the public health sector.

Compulsory licences allow local production or importation of generics from other countries, predominantly for the supply of the domestic market without the consent of the patent holder and against royalty payments to the holder to the patent. However, countries that enact a compulsory licence may face significant opposition from manufacturers defending their patent monopoly or from high income countries that have multinational pharmaceutical companies, although it is within their rights. There are important lessons to be learned from the use of TRIPS flexibilities including compulsory licensing in the procurement of low priced medicines for the treatment of HIV.

The Malaysia case shows the direct and indirect power of compulsory licensing.^22^ When Malaysia decided to issue a compulsory licence for sofosbuvir, Gilead announced the inclusion of Malaysia, Belarus, Thailand, and Ukraine in their licence agreement. Malaysia proceeded to issue a compulsory licence which permits the import of high quality generics from an Egyptian manufacturer at affordable prices.^21^ The compulsory licence enables access to generic DAA regimens of sofosbuvir and daclatasvir in 18 public hospitals in Malaysia free of charge^[[2]](#footnote-2)^. Malaysia’s inclusion in the voluntary license further enables access to other DAA combinations produced by Gilead licensees and will support treatment scale-up nationwide, including through all private and university hospitals (Figure 7). Malaysia aims to treat all 23,000 patients in the hepatitis registry and to progressively increase testing and treatment over time.^23^

The steep scale-up needed in diagnosis and treatment is a major challenge for the Malaysian response. Although the initial diagnostic step has been taken by the government to provide rapid diagnostic tests (RDT) for HCV at selected community health clinics, those diagnosed by RDT will referred to hospital specialists. The major gap in linkage to care and treatment can be addressed by decentralization of testing and treatment services at primary care and harm reduction sites, and strengthened further by reflex HCV confirmation and liver disease assessment for those tested anti-HCV positive, with capacity building and mentorship of the frontline providers.

**References**

1. The Boston Consulting Group. Road to elimination: Barriers and best practices in hepatitis C management2017.

2. Papatheodoridis GV, Hatzakis A, Cholongitas E, et al. Hepatitis C: The beginning of the end—key elements for successful European and national strategies to eliminate HCV in Europe. Journal of Viral Hepatitis. 2018; 25:6-17.

3. Martins J, Rodrigues J, Martins AP, et al. Long-Term Effect of the Portuguese Universal Access Program to New Generation Direct-Acting Antivirals for the Treatment of Hepatitis C. Journal of Hepatology. 2016; 64:S778-S9.

4. LEHC. Let's end hep C - policies. 2019 [cited 2019 March 05]; Available from: <http://www.letsendhepc.com/policies>.

5. Lim AG, Qureshi H, Mahmood H, et al. Curbing the hepatitis C virus epidemic in Pakistan: the impact of scaling up treatment and prevention for achieving elimination. International Journal of Epidemiology. 2018:dyx270-dyx.

6. Umar M, Bilal M. Hepatitis C, A Mega Menace: A Pakistani Perspective. Journal of Pakistan Medical Students. 2012; 2:68-72.

7. World Health Organization. Global report on access to hepatitis C treatment. Focus on overcoming barriers2016 October 2016.

8. World Health Organization. Pakistan tackles high rates of hepatitis from many angles. World Health Organization; 2017 [cited 2018 April].

9. Mahmood H, Qureshi H, Glass N, Averhoff F. Optimizing medicines and treatment regimens for hepatitis C patients in Pakistan. . Sao Paolo, Brazil: World Hepatitis Summit 2017; 2017 [cited 2018 April]; Available from: <http://www.worldhepatitissummit.org/docs/default-source/posters/4a_dr-hassan-mahmood.pdf?sfvrsn=2>.

10. World Health Organisation. Global Hepatitis Report 2017. <http://appswhoint/iris/bitstream/handle/10665/255016/9789241565455-engpdf;jsessionid=9DECA1FF83BC4A8C41B74E3BE2649662?sequence=1>. 2017.

11. World Health Organization. Regional action plan for viral hepatitis in the Western Pacific 2016-2020 : a priority action plan for awareness, surveillance, prevention and treatment of viral hepatitis in the Western Pacific Region. <http://iriswprowhoint/handle/106651/13141>. 2016.

12. World Health Organization. Hepatitis B Control - Country Profile 20152015.

13. Hennessey K, Mendoza-Aldana J, Bayutas B, Lorenzo-Mariano KM, Diorditsa S. Hepatitis B control in the World Health Organization's Western Pacific Region: Targets, strategies, status. Vaccine. 2013; 31:J85-J92.

14. Howell J, Van Gemert C, Lemoine M, Thursz M, Hellard M. Overview of hepatitis B prevalence, prevention, and management in the Pacific Islands and Territories. J Gastroenterol Hepatol. 2014; 29:1854-66.

15. Olafsson S., Tyrfingsson T., Runarsdottir V., et al. Treatment as Prevention for Hepatitis C (TraP Hep C) – a nationwide elimination programme in Iceland using direct‐acting antiviral agents. Journal of Internal Medicine. 2018; 283:500-7.

16. Scott N, Olafsson S, Gottfreethsson M, et al. Modelling the elimination of hepatitis C as a public health threat in Iceland: A goal attainable by 2020. J Hepatol. 2018; 68:932-9.

17. Raihan R. Hepatitis in Malaysia: Past, Present, and Future. Euroasian Journal of Hepato-Gastroenterology. 2016; 6:52-5.

18. Gilead. Chronic hepatitis C treatment expansion, generic manufacturing for developing countries, February 2016. Gilead; 2016 [updated February 2016; cited 2018 April]; Available from: <http://www.gilead.com/~/media/>ﬁ les/ pdfs/other/hcv%20generic%20agreement%20fast%20facts%20021616.pdf

19. UNITAID, World Health Organization. Technology and market landscape: Hepatitis C medicines: World Health Organization2017 August 2017.

20. pharmaceuticals G. Chronic Hepatitis C Treatment Expansion: Generic Manufacturing for Developing Countries. 2015; Available from: <http://www.gilead.com/~/media/Files/pdfs/other/HCVGenericAgreementFactSheet.pdf>.

21. Treatment Action Group. TAG Applauds Malaysian Government's Decision to Make Generic form of Life-Saving Hep C Cure. 2017 [updated 20 September 2017; cited 2018 18 April]; Available from: <http://www.treatmentactiongroup.org/content/tag-applauds-malaysian-governments-decision-make-generic-form-life-saving-hep-c-cure>.

22. Intellectual Property Watch. Malaysia Inclusion In Gilead Voluntary Licence – A Product Of Compulsory Licence Pressure. 2017 [16 Feb 2018]; Available from: <https://www.ip-watch.org/2017/08/24/malaysia-inclusion-gilead-voluntary-licence-product-compulsory-licence-pressure/>.

23. Loh Foon Fong, Clarissa Chung. Free Hepatitis C treatment for all. The Star Online2018 [updated 20 March 2018; cited 2018 13 April]; Available from: <https://www.thestar.com.my/news/nation/2018/03/20/free-hepatitis-c-treatment-for-all-2000-patients-need-not-pay-at-government-hospitals-this-year/>.

24. Schweitzer A, Horn J, Mikolajczyk RT, Krause G, Ott JJ. Estimations of worldwide prevalence of chronic hepatitis B virus infection: a systematic review of data published between 1965 and 2013. Lancet. 2015; 386:1546-55.

25. Polaris Observatory Collaborators. Global prevalence, treatment, and prevention of hepatitis B virus infection in 2016: a modelling study. The lancet Gastroenterology & hepatology. 2018; 3:383-403.

26. Gower E, Estes C, Blach S, Razavi-Shearer K, Razavi H. Global epidemiology and genotype distribution of the hepatitis C virus infection. J Hepatol. 2014; 61:S45-57.

27. Blach S, Zeuzem S, Manns M, et al. Global prevalence and genotype distribution of hepatitis C virus infection in 2015: a modelling study. The Lancet Gastroenterology & Hepatology. 2017; 2:161-76.

28. Younossi ZM, Park H, Dieterich D, et al. The value of cure associated with treating treatment-naive chronic hepatitis C genotype 1: Are the new all-oral regimens good value to society? Liver Int. 2017; 37:662-8.

29. Mohamed R, Shabaruddin FH, Dahlui M AA, SA M. Estimated 5-year acquisition cost of direct acting antiviral (DAA) for the treatment of hepatitis C in Malaysia in 2018 to 2022. Hepatology International. 2018:S181–661.

30. Chikovani I, Ompad DC, Uchaneishvili M, et al. On the way to Hepatitis C elimination in the Republic of Georgia—Barriers and facilitators for people who inject drugs for engaging in the treatment program: A formative qualitative study. PLOS ONE. 2019; 14:e0216123.

31. Cooke GS, Andrieux-Meyer I, Applegate TL, et al. Accelerating the elimination of viral hepatitis: a Lancet Gastroenterology & Hepatology Commission. The Lancet Gastroenterology & Hepatology. 2019; 4:135-84.

32. Hecht R, Hiebert L, Spearman WC, et al. The investment case for hepatitis B and C in South Africa: adaptation and innovation in policy analysis for disease program scale-up. Health Policy Plan. 2018; 33:528-38.

33. Nayagam S, Thursz M, Sicuri E, et al. Requirements for global elimination of hepatitis B: a modelling study. Lancet Infect Dis. 2016; 16:1399-408.

34. Nayagam S, Chan P, Zhao K, et al. Poster Presentations: Posters Friday, 15 April 2016: EU and public health. FRI-431: Investment Case for a Comprehensive Package of Interventions against Hepatitis B in China. Journal of Hepatology. 2016; 64:S469.

35. Lu J, Xu A, Wang J, et al. Direct economic burden of hepatitis B virus related diseases: evidence from Shandong, China. BMC health services research. 2013; 13:37.

36. Mbituyumuremyi A., Ilo Van Nuil J., Umuhire J., et al. Controlling hepatitis C in Rwanda: a framework for a national response. Bulletin Of The World Health Organization. 2018; 96:51-8.

37. Popping S, Bade D, Boucher C, et al. The global campaign to eliminate HBV and HCV infection: International Viral Hepatitis Elimination Meeting and core indicators for development towards the 2030 elimination goals. J Virus Erad. 2019; 5:60-6.

38. Doyle JS, Scott N, Sacks-Davis R, et al. Treatment access is only the first step to hepatitis C elimination: experience of universal anti-viral treatment access in Australia. Alimentary pharmacology & therapeutics. 2019; 49:1223.

39. Ahmad K. Pakistan: a cirrhotic state? The Lancet. 2004; 364:1843-4.

## Supplementary tables

Table 5: Country characteristics

|  | **Portugal** | **Pakistan** | **Fiji** | **Iceland** | **Malaysia** |
| --- | --- | --- | --- | --- | --- |
| **Population total (million, 2017)** | 10.29 | 197.02 | 0.88 | 0.34 | 31.62 |
| **Life expectancy at birth (years)** | 81 | 66 | 70 | 82.5 | 75 |
| **GNI per capita (US$)** | 19,820 | 1,580 | 4,970 | 60,830 | 9,6500 |
| **HBsAg positive population (%)** | 107,597 (1.02%) ^24^ | 4.77m (2.76%) | 38,400 (4.8%)^24^ | 448 (0.14%)^24^ | 291,000 (0.9%)^25^ |
| **HCV-RNA positive population (%)** | 82,640 (0.8%)^1^ | 7.04m (5.6%)^26^ | 1,000 (0.1%)^27^ | 1,100 (0.3%)^28^ | 380,000 (2.5%)^29^ |

Table 6: Challenges and avenues for political commitment*

| **Country** | **Initial obstacles and remaining challenges** | **How political commitment has been achieved** |
| --- | --- | --- |
| **Georgia** | **Initial obstacles:**   - Suboptimal alignment of program development/preparation and implementation – doctors were only informed of the commencement date one week prior to roll-out - Intensive advertisement in the media and enthusiasm for treatment uptake among patients led to bottlenecks, creating problems with patient flow and wait lists.^30^ - Training for healthcare workers only provided after the program launched; however, since then doctors have received continuous technical support   **Remaining challenges:**   - No estimate of the economic burden of viral hepatitis ^31^ - HBV treatment not on National Essential Medicines List (NEML) or government subsidised^31^ - High prevalence of injection drug use (IDU) ^31^ - Punitive drug laws, discrimination and stigma remain major barriers to HCV service access - Criminal responsibility for personal drug use 🡪 high levels of incarceration among people who inject drugs (PWID) and high rates of HCV in prisons - Limited access to OST in prisons - Harm reduction programmes hampered by punitive drug laws - Access to HCV care still requires out-of-pocket spending for many PWID (particularly in remote areas, as HCV care centres not equitably distributed)^30^ | - Joint efforts of civil society and NGOs   🡪 Substantial engagement and advocacy on behalf of civil society organisations involved in harm reduction   - Strong political will of the state authorities - Financial and technical support of international donors and industry to initiate world-first hepatitis C elimination program - 2011 introduction of HCV treatment in Georgia through a program supported by the Global Fund - Crucial role of the media in advertising the Elimination program; “a national pride”^30^ - Georgia as a “hepatitis C elimination champion” |
| **South Africa** | **Remaining challenges:**   - No estimate of the economic burden of viral hepatitis ^31^ - No free HBV birth dose - Third dose coverage <90% - HCV DAAs on NEML or government subsidised (not yet in place) - Free DAAs for nationals (not yet in place)^31^ - Perinatal HBV transmission major barrier to elimination - Substantial stigma and discrimination associated with viral hepatitis and punitive drug laws - National plan developed but budget allocation outstanding^32^ - Limited resources and fiscal pressures are a barrier to implementing the full national plan - Non-financial barriers include shortage of trained health workers; both at the lower and upper levels of the health system^32^ - Bottlenecks and delays in regulatory approval of DAAs are key barriers to large-scale treatment - HCV DAAs not yet procured at low cost | Formation of a South Africa Viral Hepatitis Working Group involving:   - South Africa National Department of Health - South African leading clinician advocates - Expert disease modellers - Senior health economists - (Pharos Global Health Advisors)   Successful elements:   - Linking development of national clinical guidelines with a 5-year action plan and longer-term modelling of scale-up - Early engagement and collaboration with health ministry leadership discussing emerging results - Joint health-finance sector consultative discussion |
| **Scotland** | **Remaining challenges:**   - (UK) **–** No reliable estimate of economic burden of viral hepatitis - Limited free birth dose vaccination - (UK) – No population-level coverage of free HBV vaccinations | - Well-developed linked data systems provide comprehensive epidemiological information on HCV to support policy initiatives, monitoring and evaluation |
| **Brazil** | **Remaining challenges:**   - No reliable estimate of economic burden of viral hepatitis^31^ - Third HBV dose vaccination coverage <90%^31^ - Providing equitable access to health across varied geographical regions remains a challenge (rural vs urban) - Major geographical, social and economic disparities - Limited access to specialists who can provide DAA treatment (currently available in only a few centres in Brazil) 🡪 long delays between diagnosis and treatment initiation^31^ - Low diagnosis rates and huge losses to follow-up | - Brazil’s image as an international “viral hepatitis champion” - Strong commitment thanks to early successes in HIV response - Substantial advocacy on behalf of civil society organisations helped to sustain political commitment after initial roll-out of the plan |
| **China** | **Remaining challenges:**   - National plan/strategy not yet fully developed - No reliable estimate of economic burden of viral hepatitis (in progress^33-35^) - Publicly funded screening programmes not yet implemented (in progress) - Resistance from specialist doctors to treat pregnant women for HBV - HCV DAAs on NEML or government subsidised (not yet in place) - No free DAAs for nationals - Few DAAs have been approved and their high cost has precluded them from wide coverage in basic insurance health programmes. Few provinces have included DAAs in list of meds for reimbursement - Stigma & discrimination must be overcome to increase diagnosis rates; e.g. through policy and education - Lack of awareness among public and healthcare workers that there is effective treatment available - Challenging to identifying those diagnosed (low reported rates of cases hide the millions of people living with viral hepatitis) - Exclusion of viral hepatitis related cirrhosis and liver cancer from cancer burden leads to under-representation of viral hepatitis-related morbidity and mortality | - Studies on return on public sector investment in HBV prevention and treatment, demonstrating that money spent on HBV will save money over a 15 year time period   🡪 helped develop a policy for hepatitis control   - Strong, effective public-private partnerships were catalytic to policy development |
| **Egypt** | **Initial obstacles:**   - Huge numbers of patients requiring treatment - Need for an electronic database - Need for initial treatment prioritisation   **Remaining challenges:**   - Limited harm reduction programs - Birth dose vaccination not yet free for all - Identifying chronic HCV patients is an ongoing challenge due to increasing costs of diagnosis (no generic / locally produced tests; national guidelines require multiple tests) - Application of guidelines for blood safety, injection safety and strict infection control yet to be applied nationally | - Availability of large-scale epidemiological data drove sustained societal pressure for state-sponsored treatment - Ability to achieve reduced drug-prices |
| **Rwanda** | **Initial obstacles:**   - Development of local viral hepatitis guidelines based on international consensus guidelines without sufficient consideration of the local context - The guidelines thus recommended unavailable/unaffordable viral hepatitis management (due to lack of skilled staff and insufficient resources for implementation) - These impractical guidelines have since been abandoned and are being overhauled   **Remaining challenges:**   - Limited reliable national data (in progress) - No estimate of economic burden of viral hepatitis - No harm reduction programmes (in progress) - No free HBV birth dose - Limited free DAAs for nationals - Limited access to diagnostics (access, cost) - Perinatal HBV transmission a major barrier to elimination - Punitive drug laws, discrimination and stigma remain major barriers to viral hepatitis service access - Shortage of healthcare workforce - Fiscal constraints challenge full funding of hepatitis programs; leading to continued out-of-pocket spending for the public^36^ - Further decentralisation of care from referral and provincial hospitals to district hospitals and primary care needed to improve equity - Coverage of diagnostic capacity can increase through procurement and distribution of rapid diagnostic tests | - Evidence of viral hepatitis-related disease burden exceeding deaths attributable to HIV has evoked strong political will to eliminate viral hepatitis ^37^ - Government commitment towards health equity and universal health coverage |
| **Australia** | **Remaining challenges:**   - Barriers include linkage to care for HBV (only 57% diagnosed, 13% linked to care; 5% on treatment) (2012 numbers) - Lack of awareness of the risks of HBV infection among the public and healthcare practitioners and inadequate guidelines - Declining numbers of people accessing DAA treatment ^38^ - Shortage of healthcare practitioners in remote areas and primary care - High rates of incarceration among PWID due to restrictive drug policies - No needle and syringe programs in prisons | - Evidence of viral hepatitis-related disease burden exceeding deaths attributable to HIV has evoked strong political will to eliminate viral hepatitis ^37^ - Government commitment towards health equity and universal health coverage |
| **Additional case studies** | | |
| **Portugal** | **Remaining challenges:**   - Changes in government revoked decision to centralise HCV treatment payment procedures leading to DAAs being funded the same as other drugs and resulting in waiting periods of >6 months in most hospitals - No HCV treatment in prisons; accessing hospitals one-by-one - National action plan lacks clear goals, actions or resources - Prevention, testing & diagnosis activities must improve as more than half of estimated patients remain undiagnosed (conservative estimate) | - Efforts of civil society and academic stakeholders have resulted in a consensus that focusing on policies for HCV elimination and prevention, financing, access models, a national action plan, and a central patient registry is necessary Policy-change as a source of blood-borne infection control (decriminalisation of drug consumption) - Piggy-backing on existing HIV programs and services enabled viral hepatitis-focused services without requiring the development of a fully new infrastructure or network |
| **Pakistan** | **Remaining challenges:**   - National plan/strategy not yet fully developed - No reliable estimate of economic burden of viral hepatitis (in progress) - Limited harm reduction programs - No free birth dose - Third dose vaccination coverage still below 90% - Publicly funded screening programmes not yet implemented (in progress) - DAAs for nationals not yet free for all - HCV-related stigma & discrimination 🡪 must be overcome to increase diagnosis rates - Lack of awareness among public and healthcare workers that viral hepatitis is not a death sentence - High use of therapeutic injections , reuse of syringes, and unsafe waste management^39^ (iatrogenic transmission) - Limited resources to implement comprehensive viral hepatitis strategy | - Civil society advocacy, using evidence of disease burden to garner political support - Pakistan Kidney and Liver Institute and Research Center “demonstrated the seriousness of the situation” (the impact of hepatitis C on morbidity and mortality) to the Chief Minister of Punjab - Chief Minister of Punjab took personal interest and pledged full support, which included new policies for the prevention of hepatitis^8^ |
| **Fiji** | **Remaining challenges:**   - No national strategy/plan in place^30^ - No estimate of economic burden related to viral hepatitis - No harm reduction programmes - Publicly funded screening programs not fully developed - HBV treatment on NEML or government subsidised (not yet in place) - HCV DAAs not subsidised - No free DAAs for nationals**^30^** - Insufficient resources to implement interventions - Infrastructural challenges include geographical isolation; limited access to antenatal care and attended births; inadequate vaccine supplies and cold chain systems; lack of skilled medical staff |  |
| **Iceland** | **Remaining challenges:**   - A small group of patients remain who have been difficult to engage and retain in care - Immigration; asylum seekers, foreign prisoners with pre-existing infections - Patients at risk of infection and reinfection (MSM, people who inject drugs and share equipment) |  |
| **Malaysia** | **Remaining challenges:**   - Costed national plan/strategy not yet fully developed (in progress) - Limited integration of hepatitis testing and linkage at harm reduction sites (in progress) - No publicly funded screening programmes - Limited access of free DAAs for nationals (in progress) - Stigma & discrimination 🡪 must be overcome to increase diagnosis rates - Lack of awareness among public and healthcare workers that viral hepatitis is not a death sentence - Harsh drug law enforcement leads to high incarceration rate among PWID and presents a barrier to harm reduction service access and HCV diagnostics and treatment | - Providing data on the epidemiology, disease burden and economic impact of hepatitis has garnered government commitment to prioritise the hepatitis agenda - Multi-stakeholder collaborations through campaigns (e.g. in conjunction with World Hepatitis Day) to increase hepatitis awareness, advocate and focus on the screening and treatment scale-up needed to meet the 2030 targets - Strong political leadership, civil society advocacy and media engagement |

**as identified by the Lancet Commission on “Accelerating the elimination of viral hepatitis”* ^31^ *and country experts, unless otherwise referenced*

1. This centralised payment procedure was overturned in 2018 which has led to growing discrepancies in terms of waiting time (from test to treatment) between different hospitals. Currently, waiting time can go from 1 to 12 months. [↑](#footnote-ref-1)
2. See publicly available database on TRIPS flexibilities: http://tripsflexibilities.medicineslawandpolicy.org/ [↑](#footnote-ref-2)
